# Textile dual-band NFC-A4WP (13.56–6.78 MHz) combiner for wireless energy and data transmission for connected clothing

**DOI:** 10.1038/s41598-023-31832-0

**Published:** 2023-04-06

**Authors:** Baptiste Garnier, Philippe Mariage, François Rault, Cédric Cochrane, Vladan Koncar

**Affiliations:** 1 Ecole Nationale Supérieure des Arts et Industries Textiles, Roubaix, France; 2 CNRS, Centrale Lille, Univ. Polytechnique Hauts-de-France, UMR 8520 - IEMN, Univ. Lille, 59000 Lille, France

**Keywords:** Devices for energy harvesting, Electrical and electronic engineering, Magnetic properties and materials

## Abstract

An original fully textile combiner is proposed to power supply sensors close to a body with only one centralized source of energy like a smartphone, for instance. A solution is provided for taking into account the requirements of an industrial production process that need to minimize needle movements during an embroidery process. Moreover, the paper shows how to support several wireless power transmission standards that already exist, i.e. NFC and A4WP, or will exist to satisfy the tremendous needs of energy for distributed systems in the IoT domain. In this paper, a new textile-based flexible wireless system enabling communication and energy harvesting is proposed. Analytical, numerical, and experimental studies have been conducted to demonstrate that the structure has two resonant frequencies at 6.8 MHz and 13.6 MHz, which make it suitable for NFC and A4WP standards. Moreover, the losses caused by the system are 2.76 dB and 1.91 dB for A4WP and NFC, respectively. The results are successively presented to highlight the specificities of such textile multi-coils combiners. A method for gaining a resonant structure without any solid electronic component is explained.

## Introduction

The connected textiles recent development enables the rise of data and energy transmission within clothing. Indeed, the embedded sensors in our cloth need a power supply to work and transmit data. Near-field Communication (NFC) technology is a solution to centralized energy sources and data storage, especially with the battery and memory capacity improvement. Progress in electronics has already enabled to use of wireless power transfer (WPT) between wearable small devices and smartphones, NFC technology equipped^1,2^. Some small IoT devices such as physiological sensors may require to be sometimes only power supplied, eventually by a technology standardized by the A4WP (Alliance for wireless power) recently re-named Airfuel (Air Fuel Alliance), knowing that the Qi technology is an alternative standard, that is already available on the market^3–5^. The latter technology has not been retained in the present work as it runs at very weak frequencies around 300 kHz which would restrict its usage to perfectly aligned and identical antennas for the transmitter and receiver located near the combiner. It is to be noticed furthermore that the frequency of the A4WP standard is exactly half of that of the NFC frequency.

Numerous energy sources can be used to power supply e-textile, but smartphones are today equipped with NFC antennas and can store, process, and send a large amount of data. Consequently, they are particularly adapted to the power supply and connect smart textiles to a different network. For example, an energy transmission system has been developed to power supply smart textiles from bicycle mechanical energy^6^. Jiang et al. have also developed a textile NFC antenna with silver-plated yarns able to transmit data even under flexions^7^. More recently, Rongzhou Lin et al. have also published a study presenting an integral textile NFC transmission system embroidered on a garment^8^. It aims to monitor real-time physiological parameters in a nomadic way, like during running. However, the device needs a few rigid electric components to work and its operating frequency is not adjusted to 13.56 MHz. Another study on textile NFC antennas focused on the resonant frequency aspect^9^. It shows that the embroidered antenna can be realized only with textile materials and processes, and its resonant frequency can be adjusted to the NFC technology. Finally, the association of several textile antennas can create new devices, called “combiners”, enabling to transfer of a 13.56 MHz magnetic field through a textile surface^10^. Unfortunately, this kind of structure includes a soldering point to close the circuit, which produces a weakness in the device. There are also textile near-field multibody area network structures used for on-body communication developed by metamaterials built from arrays of discrete, anisotropic magneto-inductive elements^11^. Unlike the results published in recent articles on that topic, our work proves the possibility to communicate and transfer energy following two different wireless standards (NFC and A4WP) without any traditional electronic component. Only the textile material and processing techniques are used to design and produce the antenna and combiner.

Compared to the previous structure^10^, the soldering point has been replaced by a textile capacitor. First, a theoretical study of the structure highlights the presence of two resonant frequencies reliant on the antenna's intrinsic resonance and the value of the new textile capacity, respectively. Second, simulations have been conducted to evaluate the structure's electrical responses. Third, the transmission coefficient S_21_ has been measured to identify the experimental resonant frequencies. Finally, a practical application has been realized, as a proof of concept.

## Materials and methods

### Textile dual-band near field multiple combiners

**Figure 1:**
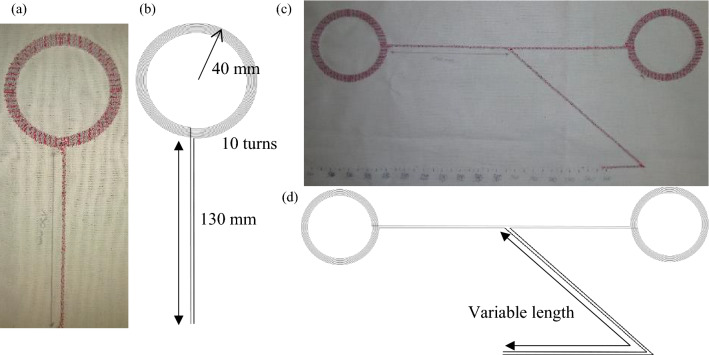
(**a**) Photography of the antenna and (**b**) its diagram. (**c**) Photography of the two antennas textile dual-band (NFC-A4WP) combiner and (**d**) its diagram. Figure 1(**b**) and (**d**) were carried out using Inkscape software v1.2.2 (https://inkscape.org/).

The textile dual-band (NFC-A4WP) combiner enables to transfer of energy and data across clothing at two different frequencies, 13.56 MHz and 6.78 MHz, by using successive magnetic induction couplings. The device is composed of several antennas that can be used as a transmitter or a receiver. All antennas are identical and composed of a 40 mm, 10 turns coil, connected to a 130 mm transmission line, as shown in Fig. 1a, b. They are associated symmetrically, to form a circuit composed of a single current line with an embroidering process. Two current lines, with variable lengths, are added to the embroidery start and end. A picture and a diagram of the device are shown in Fig. 1c, d. The current line is formed by using three overlapped textile conductive yarns Datatrans, from Tibtech Company.

The antenna's electrical characteristics are already known from previous studies^9,10^. A Textile dual-band (NFC-A4WP) combiner with two of these kinds of antennas has been prototyped to evaluate its transmission coefficients. Also, the length of the current lines at the start and the end of the embroidery and forming a section of the parallel transmission line is variable to study its impact on the resonant frequencies and the transmission coefficients.

### Theoretical electrical characteristics

**Figure 2:**
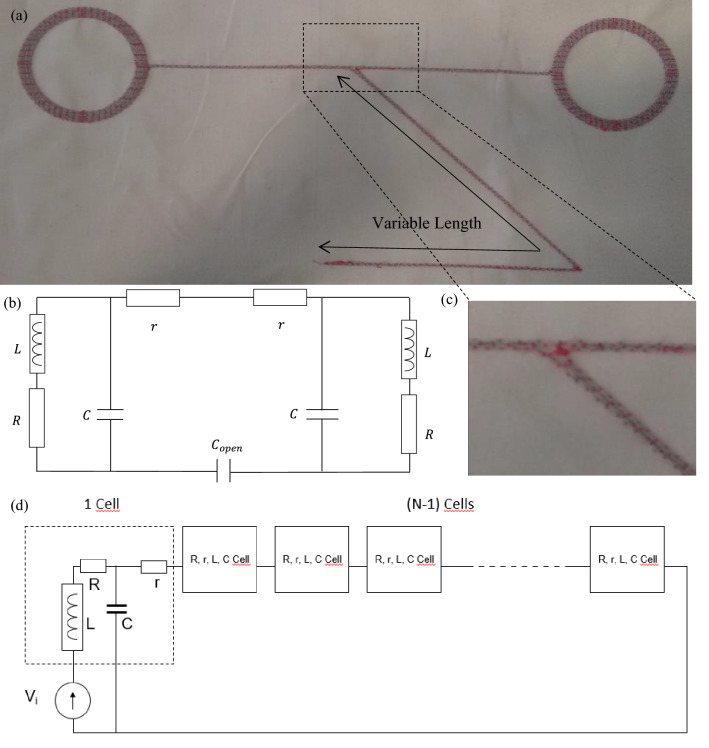
(**a**) The two antennas textile dual-band (NFC-A4WP) combiner, (**b**) its electric diagram and (**c**) a zoom on the opening capacity Copen. (**d**) Case of an N-coils combiner (cells) inductively excited (inductive voltage source).

Textile dual-band (NFC-A4WP) combiner and its electric diagrams are presented in Fig. 2a, b, with L as the inductance, C as the capacity of one antenna, R as the coil resistance, and r as the transmission line resistance. C_open_ is the capacity of the additional open-ended section of the transmission line, called AOETL in the rest of the paper, with a variable length forming the start and end of the embroidery. Assuming one of the coil antennas is excited by inductive coupling, the circuit impedance viewed by the induced voltage source (not shown in Fig. 2) can be expressed by the Eq. (1) where ω is the pulsation of the sinusoidal signal and j is the square root of − 1.

1 $$Z_{viewed by the induced voltage source} = jL\omega + R + \frac{1}{{jC\omega + \frac{1}{{2r + \frac{1}{{jC_{open} \omega }} + \frac{R + jL\omega }{{1 + jRC\omega - LC\omega^{2} }}}}}}$$

Although Fig. 2b shows a 2-coils combiner, the following analytical study gives more generalized equations adapted to an N-coils combiner (1 input, (N-1) outputs) as depicted in Fig. 2d. Each antenna is modelled by a cell with r, R, L, and C parameters already defined in the 2-coils combiner case. The left antenna is inductively coupled to a not shown transmitter resulting in the presence of a voltage source Vi. Thus, the impedance Z_i_ viewed by the source is given by the following equation:

$${Z}_{i}=jL\omega +R+\frac{1}{jC\omega +\frac{1}{Nr+\frac{1}{j{C}_{open}\omega }+\left(N-1\right)\frac{R+jL\omega }{1+jRC\omega -LC{\omega }^{2}}}}$$

The resonance conditions are obtained when the imaginary part of the impedance vanishes. The C_open_ capacity being in series with a sum of parallel resonant circuits (coils) may bring a new resonant frequency in addition to the initial resonance coming from the antennas^12^, except for two simplified scenarios that have been analytically studied. First, the case where the circuit resonates only at the proper frequency of each antenna. Then, the case where the capacity of C_open_ is very small compared to the specific antenna’s capacities. In each case, the conditions that enable the influence of this opening to be neglected will be emphasized. They are obtained when the impedance fed into the circuit is negligible compared to the modulus of the impedance, denoted Z’, of the N-1 antennas connected in series with it.

The impedance provided by C_open_ and by the (N-1) antennas connected in series are respectively given by the following expressions:

$$\left| {\frac{1}{{jC_{{{\text{open}}}} \omega }}} \right| = \frac{1}{{C_{open} \omega }}$$

$$\begin{aligned} \left| {Z^{\prime}} \right| & = \left( {N - 1} \right)\left| {\frac{{R + j\omega \left( {L - L^{2} C\omega^{2} - R^{2} C} \right)}}{{1 + \omega^{2} \left( {C^{2} L^{2} \omega^{2} + R^{2} C^{2} - 2LC} \right)}}} \right| \\ & = \left( {N - 1} \right)\left| {\frac{{\sqrt {R^{2} + \omega^{2} \left( {L - L^{2} C\omega^{2} - R^{2} C} \right)^{2} } }}{{1 + \omega^{2} \left( {C^{2} L^{2} \omega^{2} + R^{2} C^{2} - 2LC} \right)}}} \right| \\ \end{aligned}$$

At the circuit proper resonance (if N antennas are identical), there is $LC\omega^{2} = 1$So

$$\left| {Z^{\prime}} \right| = \left( {N - 1} \right)\left| {\frac{{\sqrt {R^{2} + \frac{1}{LC}\left( {R^{2} C} \right)^{2} } }}{{1 + \frac{1}{LC}\left( {R^{2} C^{2} - LC} \right)}}} \right|\; = \;\left( {N - 1} \right)\left| {\frac{{\sqrt {R^{2} + \frac{C}{L}R^{4} } }}{{1 + R^{2} \frac{C}{L} - 1}}} \right| = \left( {N - 1} \right)Q^{2} R\sqrt {1 + \frac{1}{{Q^{2} }}} \approx \left( {N - 1} \right)Q^{2} R if Q > > 1$$

At that resonant frequency, there is also: $\frac{1}{{C}_{open}\omega }=\frac{1}{{C}_{open}\frac{1}{\sqrt{LC}}}=\frac{\sqrt{LC}}{{C}_{open}}$

C_open_ will not influence the value of the resonant frequency if its associated impedance is far less than the value of the impedance of the whole (N-1) antennas. That is:

$$\frac{\sqrt{LC}}{{C}_{open}}\ll \left(N-1\right){Q}^{2}R$$

This condition results in the following requirement:

$${C}_{open}\gg \frac{C}{\left(N-1\right)Q}$$

In the other particular case where *C*_open_ is extremely low beside the antenna capacity (${C}_{open} << C$), the total impedance of the circuit is given by the following expression, ε being the infinitesimal value of the equivalent impedance of the (N-1) antennas.

$$Z=jL\omega +R+\frac{1}{jC\omega +\frac{1}{Nr+\frac{1}{j{C}_{open}\omega }+\varepsilon }}$$

Assuming that ε and Nr are both negligible, the impedance Z is given by the expression:

$$Z\approx jL\omega +R+\frac{1}{jC\omega +j{C}_{open}\omega }=R+j\left[L\omega -\frac{1}{\left(C+{C}_{open}\right)\omega }\right]$$ $$Z\approx R+j\left(L\omega -\frac{1}{C\omega }\right)$$ because $${C}_{open} \ll C$$

The resonance will appear at: ${f}_{0}=\frac{1}{2\pi \sqrt{LC}}$

### Prototype fabrication

Every textile dual-band NFC-A4WP combiner was designed with the GiSBac software and produced with an industrial embroidery machine JF-0215–495 from the ZSK Company. The structure has been realized on cotton fabric with a Datatrans conductive yarn from Tibtech Company, as a bobbin thread, and with a basic hosiery cotton yarn, as the upper thread. The current line composing the circuit is formed by overlapped Datatrans conductive yarns.

### Characterization

**Figure 3:**
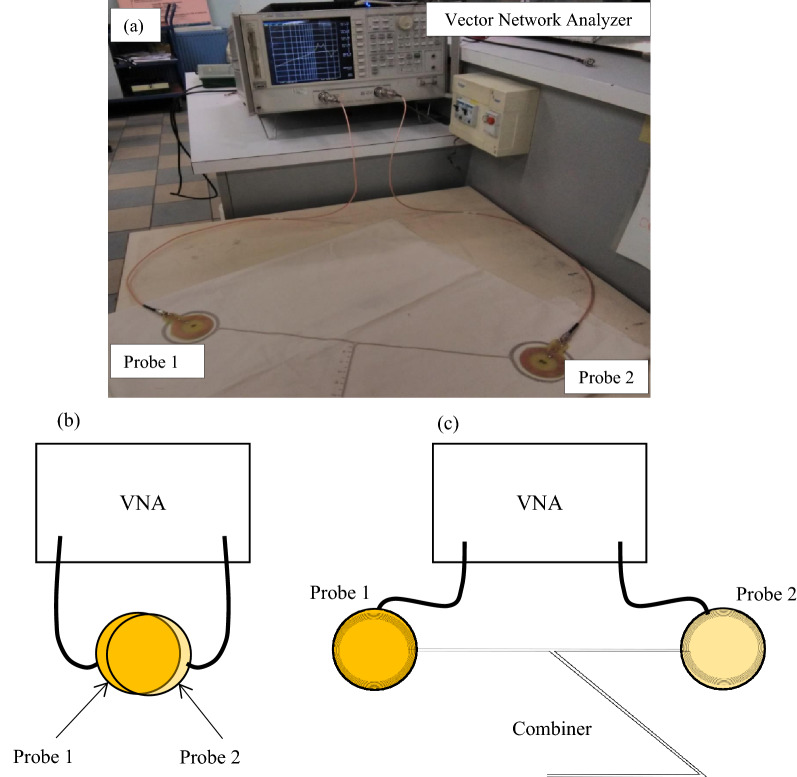
(**a**) Photo of the combiner S_21_ parameter measurement experimental setup. (**b**) Diagram of the direct transmission between the two probes. (**c**) Diagram of the dual band combiner’s S_21_ parameter measurement experimental setup. Figure 3(**b**) and (**c**) were carried out using Microsoft PowerPoint 2016 16.0.4266.1001 (https://www.microsoft.com/en-ca/microsoft-365/powerpoint).

All the characterizations have been conducted under the conditions of a temperature of 21 °C and relative humidity of 65% of our standardized laboratory. The Vector Network Analyzer used was an Agilent 8753S. The scattering parameters measurement has been realized thanks to a probe PCB antenna connected with a 1 m coaxial cable. The used printed probe coil antennas have a 22 mm mean radius, a conductive tracks width of 0.6 mm and 8 turns. They have been printed on a 1.6 mm thickness FR4 substrate. The VNA calibration has been performed with the coaxial cable to suppress their influences on the results. A photo of the combiner’s S_21_ parameter measurement experimental setup and, diagrams of the direct transmission and combiner transmission measurements are presented in Fig. 3. The electromagnetic field cartography has been realized with a ScanPhone from Luxondes Company.

## Results

### Electrical characteristics simulation

**Figure 4:**
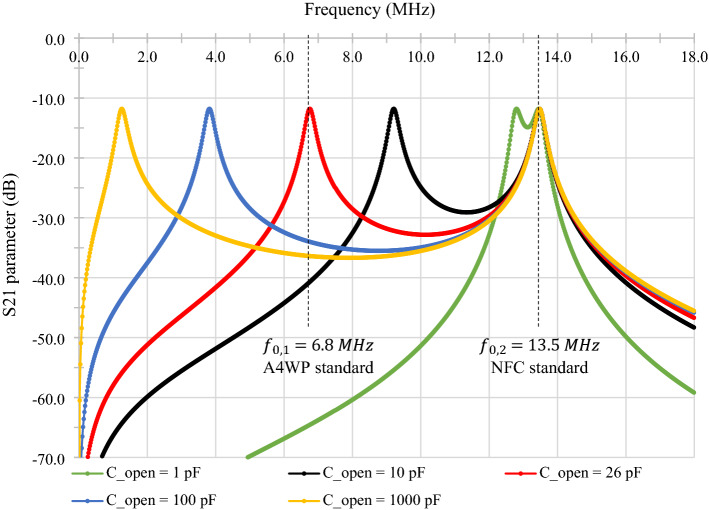
Simulations of the dual-band combiner’s S_21_ parameter for C open = [1pF, 10pF, 26pF, 100pF, 1000pF] from 1 to 18 MHz (Simulated values by LTspice).

The simulations have been realized with the LTspice software. All constant data in these simulations come from a previously published study^9^. The textile dual-band (NFC-A4WP) combiner with two antennas' electrical characteristics has also been simulated to confirm our theoretical results. The parameter values used in the simulation are $R=14.1 \Omega$, $L=7.99 \mu H$, $C=17.45 pF$. These values were been choose concerning a previous paper^9^ and have been adapted to a 40 mm, 10 turns, and 130 mm transmission line textile NFC antenna. The variable capacity ${C}_{open}$ has been fixed at $1 pF, 10 pF, 100 pF and 1000 pF$ to view its impact on the second resonant frequency. A fifth value of ${C}_{open}=26 pF$ has also been traced to highlight a dual-band structure adapted to both NFC and A4WP standards. Figure 4 shows the simulation results of the current running in the inductance of an antenna.

These simulations demonstrate the effect of the opening capacity on the appearance of a second resonant frequency in the structure. Indeed, the resonance at the highest frequency, ${f}_{\mathrm{0,2}}=13.5 MHz$, remains fixed and is equal to the resonant frequency of the antennas, which make up the combiner. In contrast, the second resonant frequency, ${f}_{\mathrm{0,1}}$ (at the lower value) is dependent on the value of the opening capacity. The higher its value is, the further the resonant frequency is from $13.5 MHz$. Finally, as shown by the former analytic development, when ${C}_{open}$ is very small compared to the capacity of an antenna ($C$), then the resonance maxima merge. This case tends to appear in Fig. 3, for C_open_ = 1pF which begins to be small compared to the value of C = 17.45pF.

### Resonant frequencies experimental results

**Figure 5:**
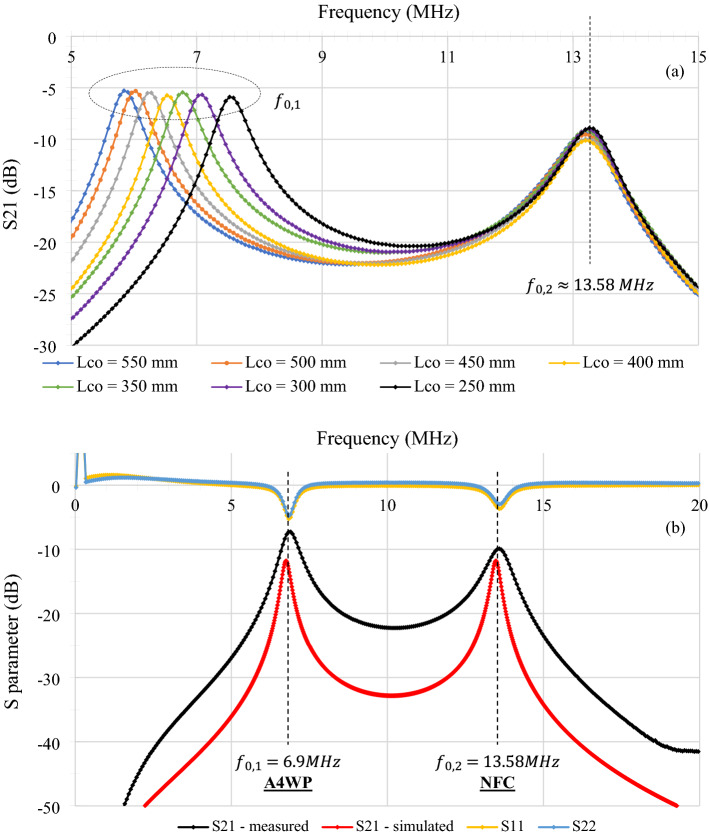
(**a**) Textile dual-band combiner’s measured S_21_ parameter with opening length ${L}_{co}$(mm) varying from 250 to 550 mm. (**b**) The NFC-A4WP adapted textile dual-band combiner’s measured and simulated S_21_ parameter from 0 to 20 MHz. The measured S_11_ and S_22_ parameters. The A4WP and NFC standard frequencies are highlighted.

The 2-coil textile dual-band (NFC-A4WP) combiner transmission coefficient (S_21_ parameter) has been measured to determine its resonant frequency according to the opening capacity ${C}_{open}$ value. To modify this value, the length of the AOETL has been reduced from 550 to 250 mm with a 50 mm interval. Figure 5a presents the results of the structure’s S_21_ parameters between two antennas inductively coupled to each coil of the combiner, where ${L}_{co}$ is defined as AOETL length. The choice has been made to use non-resonant transmitter and receiver antennas later called “probes” to characterize the ability of the combiner alone to transmit both frequencies in certain conditions. A transmission coefficient peak expresses a resonant frequency. These results highlight the presence of two resonant frequencies. The first, ${f}_{\mathrm{0,1}}$, comes from the opening capacity and varies according to its value. A length of 250 mm of ${L}_{co}$ enables it to reach a 5.8 MHz resonant frequency. The second resonant frequency ${f}_{\mathrm{0,2}}$ is stable and its value depends on the antenna's characteristics (L and C), in this case,${f}_{\mathrm{0,2}}=13.58 MHz$. When the value of ${L}_{co}$(i.e. those of the opening capacity) decreases, the first resonant frequency gets closer to the second, according to the theoretical analysis and the simulations.

These measurements have enabled us to determine the adapted length of the AOETL to create a resonant structure at NFC and A4WP frequencies, i.e. 13.56 MHz and 6.78 MHz. The realized prototype is composed of a 360 mm opening length. Its measured and simulated S_21_ parameters, and the measured S_11_ and S_22_ parameters, presented in Fig. 5b, bring out two resonant frequencies in the A4WP and NFC range: ${f}_{\mathrm{0,1}}=6.77\mathrm{ MHz}$ and ${f}_{\mathrm{0,2}}=13.58 MHz$.

### Textile dual-band (NFC-A4WP) combiner efficiency

Regarding the power transfer efficiency, the direct transmission S_21_, between the two probes when superposed without the combiner structure has also been measured to evaluate the impact of the textile dual-band NFC-A4WP combiner on the power transmission. The direct transmission S_21_ is considered the reference measure. Consequently, the relative losses $L (dB)$, produced by the structure at their resonant frequencies are the difference between the direct transmission S_21, direct_ (dB), shown in Fig. 3b, and the textile combiner S_21, combiner_ (dB), shown in Fig. 3c. The values of the transmission coefficient S_21_ (direct and via the combiner) and the relative losses L (dB) at 6.9 MHz and 13.58 MHz have been presented in Table 1. The results show relative losses of 2.76 dB and 1.91 dB at the first and second resonant frequencies, respectively. Consequently, the textile dual-band (NFC-A4WP) combiner enables to transfer of energy with minimal losses, despite the successive magnetic induction couplings between the two probe antennas.

**Table 1:** S21 parameter of the combiner and the direct transmission; and the relative losses produced by the textile dual-combiner NFC-A4WP.

Frequency	S_21_ direct (dB)	S_21_ combiner (dB)	Relative losses L (dB)
6.9 MHz	− 4.51	− 7.27	2.76
13.58 MHz	− 8.00	− 9.91	1.91

### Electromagnetic field emissions

The textile dual-band (NFC-A4WP) combiner aims to increase the operating range of the NFC and A4WP protocols through a textile surface in a fully contactless manner. However, the electromagnetic field emission has to be confined to the specific area along with the textile materials.

**Figure 6:**
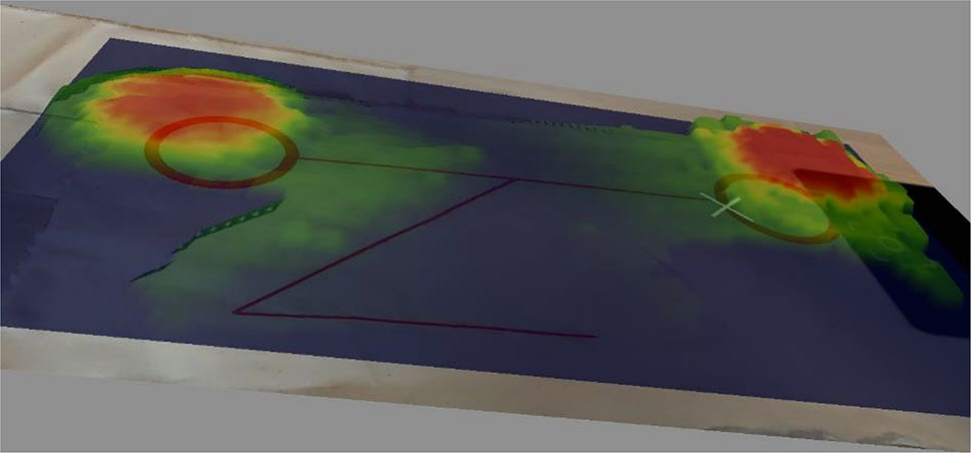
Textile dual-band (NFC-A4WP) combiner electromagnetic field emission cartography.

An electromagnetic field emissions cartography has been conducted on the prototype to locate the high emission areas and to evaluate the structure emission losses, in particular along the transmission line sections between coils and the so-called AOETL. The measurement has been carried out thanks to a ScanPhone from Luxondes^13^. The textile dual-band combiner has been power supplied by magnetic induction with 13.56 MHz and 2 Vpp signals from an HF generator. The measure of the magnetic field strength has been performed at 20 mm above the prototype. The results, presented in Fig. 6, underscore two high-emission areas, located on both coils, as expected. The cartography also shows few emission losses along the transmission line sections of the structure but remains limited.

## Proof of concept

**Figure 7:**
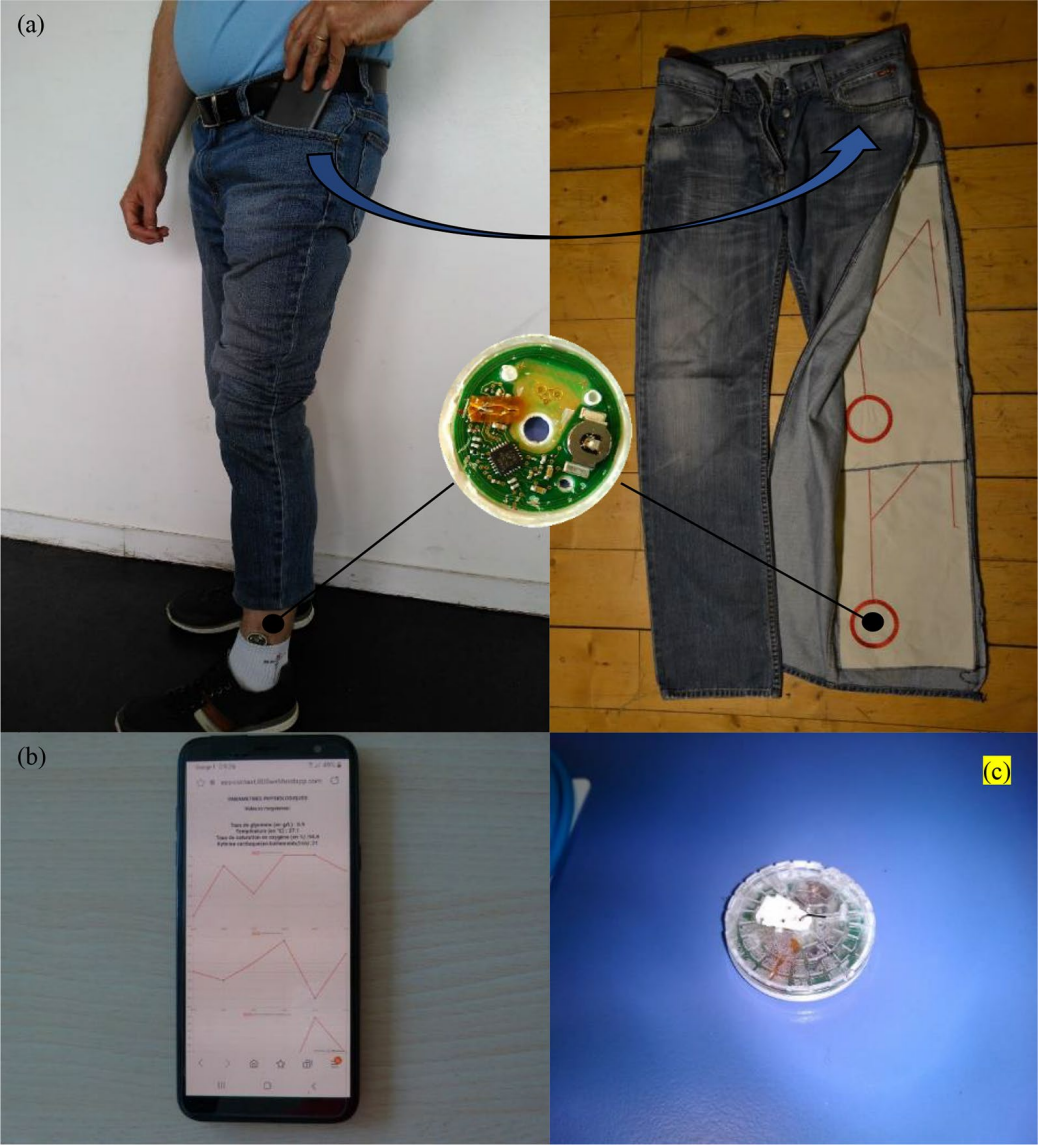
(**a**) The textile dual-band near field multiple combiner proof of concept, (**b**) the data transmitted and (**c**) the sensor.

The textile Dual-band Near Field multiple combiners aim to improve body-centric communication, which means creating power and data transfer between a smartphone (Samsung A6 with NXP TagInfo Android application) and body sensors (FreeStyle Libre, a continuous monitoring sensor from Abott Diabetes Care). To demonstrate the feasibility of the device proof of concept has been realized. The textile dual-band near field multiple combiners have been integrated into trousers to establish an electromagnetic connection (power and data) between a smartphone, placed in the pocket, and a physiological parameters sensor, placed on the ankle. Figure 7 presents the complete device, the sensor, and the data transmitted.

## Conclusion

This paper deals with the usage of a fully textile structure aimed to distribute energy around the body by using inductive resonant coupling. That energy may come from a device (e.g. smartphone or wireless power bank) that can support either the NFC or the A4WP technology. The developed structure already resonates at A4WP and NFC standard frequencies (6.78 and 13.56 MHz), but the embroidering process enables large flexibility in the operating frequency. Indeed, it depends on the structure geometry as shown in the previous equations. That means it is possible to develop a textile combiner with any resonant frequencies in the MHz range by adjusting the turn number, the radius, or the transmission line length. The entire structure can be embroidered without the need to raise the needle of the industrial machine used, this process is also repeatable and fast (5 min of production for a prototype). Consequently, the proposed method is already adapted to an industrial purpose. Although the combiner may contain several output coupling antennas, the shown prototype is limited to a 2 coils antenna combiner that serves to gain numerical modelling and measurement results pointing out the behaviour analytically forecastedcxs. Finally, losses caused by the textile dual-band near-field multiple combiners are acceptable for RFID applications, even if they can be reduced. For example, by working on impedance adaptation.

## Supplementary Information

Supplementary Information 1.

Supplementary Information 2.

Supplementary Information 3.

## Data Availability

All data generated or analysed during this study are included in this published article and its supplementary information files.

## References

[1] Park J (2017). A resonant reactive shielding for planar wireless power transfer system in smartphone application. IEEE Trans. Electromagn. Compat..

[2] Song M (2017). Wireless power transfer inspired by the modern trends in electromagnetics. Appl. Phys. Rev..

[3] Cheng L (2017). A 6.78 MHz single-stage wireless power receiver using a 3-mode reconfigurable resonant regulating rectifier. IEEE J. Solid-State Circuits.

[4] Choi J (2013). Resonant regulating rectifiers (3R) operating for 6.68 MHz resonant wireless power transfer (RWPT). IEEE J. Solid-State Circuits.

[5] Bai X (2017). A High-Efficiency 678 MHz full active rectifier with adaptive time delay control for wireless power transmission. IEEE Trans. Very Scale Integr. Syst..

[6] Wagih M (2020). Dual-receiver wearable 6.78 MHz resonant inductive wireless power transfer glove using embroidered textile coils. IEEE Access.

[7] Jiang Y (2019). e-Textile embroidered wearable near-field communication RFID antennas. IET Microwaves Antennas and Propagation.

[8] Rongzhou L (2020). Wireless battery-free body sensor networks using nea-field-enabled clothing. Nat. Commun..

[9] Garnier B (2020). Textile NFC antenna for power and data transmission across clothes. Smart Mater. Struct..

[10] Garnier B (2021). Electronic-components less fully textile multiple resonant combiners for body-centric near field communication. Sci. Rep..

[11] Hajiaghajani A (2021). Textile-integrated metamaterials for near-field multibody area networks. Nat. Electron.

[12] Kung ML (2015). Enhanced analysis and design method of dual-band coil module for near-field wireless power transfer systems. IEEE Trans. Microw. Theory Tech..

[13] https://www.luxondes.com/intro_scanphone/, 2023.

